# Airway surface liquid volume expansion induces rapid changes in amiloride-sensitive Na^+^ transport across upper airway epithelium-Implications concerning the resolution of pulmonary edema

**DOI:** 10.14814/phy2.12453

**Published:** 2015-09-02

**Authors:** Fouad Azizi, Abdelilah Arredouani, Ramzi M Mohammad

**Affiliations:** 1 Interim Translational Research Institute, Academic Health System, Hamad Medical CorporationDoha, Qatar; 2 Qatar Biomedical Research Institute, Qatar FoundationDoha, Qatar

**Keywords:** Airway epithelium, airway surface liquid, edema, Na^+^ transport, sodium pumps

## Abstract

During airway inflammation, airway surface liquid volume (ASLV) expansion may result from the movement of plasma proteins and excess liquid into the airway lumen due to extravasation and elevation of subepithelial hydrostatic pressure. We previously demonstrated that elevation of submucosal hydrostatic pressure increases airway epithelium permeability resulting in ASLV expansion by 500 *μ*L cm^−2^ h^−1^. Liquid reabsorption by healthy airway epithelium is regulated by active Na^+^ transport at a rate of 5 *μ*L cm^−2^ h^−1^. Thus, during inflammation the airway epithelium may be submerged by a large volume of luminal liquid. Here, we have investigated the mechanism by which ASLV expansion alters active epithelial Na^+^ transport, and we have characterized the time course of the change. We used primary cultures of tracheal airway epithelium maintained under air interface (basal ASLV, depth is 7 ± 0.5 *μ*m). To mimic airway flooding, ASLV was expanded to a depth of 5 mm. On switching from basal to expanded ASLV conditions, short-circuit current (*I*_sc_, a measure of total transepithelial active ion transport) declined by 90% with a half-time (*t*_1/2_) of 1 h. 24 h after the switch, there was no significant change in ATP concentration nor in the number of functional sodium pumps as revealed by [^3^H]-ouabain binding. However, amiloride-sensitive uptake of ^22^Na^+^ was reduced by 70% upon ASLV expansion. This process is reversible since after returning cells back to air interface, *I*_sc_ recovered with a *t*_1/2_ of 5–10 h. These results may have important clinical implications concerning the development of Na^+^ channels activators and resolution of pulmonary edema.

## Introduction

The conducting and respiratory airway epithelia are lined with a thin layer of liquid, known as the airway surface liquid (ASL) (Matsui et al. 2000). The composition and depth (volume) of the ASL are important for optimal mucociliary clearance in the conducting (tracheal) airway epithelium and gas exchange in the respiratory (alveolar) airway epithelium (Hollenhorst et al. 2011). Both alveolar and tracheal airway epithelia share a common active ion transport that plays an important role in regulating the depth and possibly the ionic composition of the airway surfaced liquid (ASL) (Wu et al. 1998; Widdicombe 1999; Song et al. 2009). In healthy airways, two major processes have been described: active absorption of Na^+^ and active secretion of Cl^−^. The former should reduce the volume of ASL; the latter should increase it (Wu et al. 1998). The major driving force for liquid reabsorption across airway epithelium is provided by the passive diffusion of sodium through the apical amiloride-sensitive epithelial Na^+^ channels (ENaCs) (Johnson et al. 2002; Zemans and Matthay 2004). Then, the Na^+^ ions are pumped out of the cell by the basolateral ouabain-sensitive Na^+^/K^+^-ATPase pumps (Saumon and Basset 1993; Bhattacharya et al. 1989; Miserocchi et al. 2001a; Matthay et al. 2002; Mutlu and Sznajder 2005). The transepithelial Na^+^ transport in turn generates an osmotic gradient which drives the movement of water from the apical to the basolateral side of airway epithelium (Althaus et al. 2011). This process was mainly demonstrated by the early death of *α*-ENaC knockout mice due to their inability to clear liquid from their lungs (Hummler et al. 1996). Inflammatory diseases of the distal and upper airway epithelia are characterized by plasma transudation which contributes to the increase in airway surface liquid volume (ASLV) (Atkinson and Kaliner 1995; Persson et al. 1998; Widdicombe 2002). The main process by which increased ASLV occurs involves a raise in subepithelial hydrostatic pressure (SHP) by inflammatory mediators (Basset et al. 1987; Miserocchi et al. 2001a,b), and a decrease in the epithelial barrier integrity (Saetta et al. 1991; Jeffery 1998; Persson et al. 1998). The resulting increase in epithelial hydraulic conductivity causes a bulk flow of liquid into the airway lumen (Kondo et al. 1992; Azizi et al. 1997; Widdicombe 2002). However, some inflammatory cytokines (i.e., IL-1*β* and TNF-*α*) were found to induce ASLV expansion through CFTR (Cystic Fibrosis Transmembrane conductance Regulator) activation and without causing a change in hydrostatic pressure (Baniak et al. 2012; Collawn and Matalon 2014). ASLV expansion is a hallmark of lung edema which is usually associated with an elevation of interstitial hydrostatic pressure (Bhattacharya et al. 1989; Miserocchi et al. 2001a; Mutlu and Sznajder 2005).

In our previous in vitro study (Azizi et al. 1997), elevation of SHP to 20 cm H_2_O increased bulk flow of liquid by 500 *μ*L cm^−2^ h^−1^, and similar volume flow of liquid (750–4000 *μ*L h^−1^) was measured in vivo (Saldias et al. 2001; Kaestle et al. 2007). This bulk flow corresponds to an increase in ASLV depth of ≥60 *μ*m min^−1^ from an initial depth of 5–20 *μ*m (Johnson et al. 1993; Azizi et al. 1997; Saldias et al. 2001; Tarran et al. 2001). In cultured human airway epithelial cells, active Na^+^ transport drives liquid reabsorption at a rate of 5 *μ*L cm^−2^ h^−1^ (Jiang et al. 1993). However, in the ex vivo human or rat lung, liquid reabsorption (clearance) was measured at a slower rate of 0.1 *μ*L cm^−2^ h^−1^ (Basset et al. 1987; Jiang et al. 1993; Sakuma et al. 1994). Thus, during inflammation as in severe acute lung injury or severe hydrostatic edema, the airways (e.g., alveolar airspaces) may be flooded by a large volume of liquid (Zemans and Matthay 2004). Indeed, extravascular lung water content can reach up to 15–20 mL Kg^−1^ in patients with acute respiratory distress syndrome (ARDS) in response to elevated SHP (Sibbald et al. 1985; Mitchell et al. 1992). Impairment of excess ASL clearance occurs during both hydrostatic pulmonary edema (Verghese et al. 1999; Saldias et al. 2001; Kaestle et al. 2007) and acute lung injury (Verghese et al. 1999; Mutlu and Sznajder 2005). In patients with severe hydrostatic lung edema, alveolar fluid reabsorption was found to be impaired or reduced in 62% of cases (Verghese et al. 1999). Similarly, the majority of patients with acute lung injury have impaired ASL clearance (Ware and Matthay 2001). In experimental animals, it was reported that alveolar fluid clearance decreased by 50% in rats exposed to a left atrial pressure (LAP) of 15 cm H_2_O (Saldias et al. 2001). Similarly, sheep ventilated with high LAP (24 cm H_2_O gradient) had a 30% reduction in alveolar fluid clearance (Campbell et al. 1999). The pathophysiological mechanism(s) that lead to reduced or impaired ASL clearance has not been fully elucidated. Nevertheless, an association between acute elevation of SHP and a decrease in active Na^+^ transport has been reported but without establishing clearly a cause and effect relationship between them (Saldias et al. 2001; Kaestle et al. 2007; Althaus et al. 2011; Hollenhorst et al. 2011). In our previous study (Azizi et al. 1997), only the paracellular but not the transepithelial permeability (e.g., active ion transport) was found to be directly altered by SHP. Thus, the large volume of ASL induced by SHP may be responsible for the alteration of active Na^+^ transport across (upper) airway epithelium. To test this hypothesis, we conducted an in vitro study using cultures of primary tracheal epithelial cells. Like the native healthy airway epithelium, primary airway epithelial cells maintained under air–liquid interface (ALI) culture possess a high degree of morphological differentiation and a normal active ion transport (Whitcutt et al. 1988; Kondo et al. 1991, 1993; Yamaya et al. 1992; Johnson et al. 1993). The mucosal surface of airway epithelial cells grown under ALI is not completely dry; there is a thin film of liquid with a depth of approximately 7 ± 0.5 *μ*m (Basal ASLV) (Tarran et al. 2001). To mimic (the upper) airway flooding, ASLV was increased to some 5 mm deep by adding culture medium to the mucosal surface of airway epithelial cells (ASLV expansion).

The magnitude of active ion transport processes across airway epithelium can be measured in vitro using Ussing chambers in which current is passed across the tissue to bring the transepithelial potential difference to zero (Ussing and Zerahn 1951; Koefoed-Johnsen and Ussing 1958).The current needed to achieve this is known as the short-circuit current (*I*_sc_), and is equal to the sum of all active ion transport processes acting across the tissue. The exact nature of the transport processes generating the *I*_sc_ can be determined with radioisotopes or by using pharmacological blocking agents. It is already established that ALI (Basal ASLV) promotes a high *I*_sc_ largely by stimulating active Na^+^ transport across airway epithelial cells (Kondo et al. 1991, 1993; Johnson et al. 1993; Yamaya et al. 1993). In this study, we have examined the time course by which ASLV expansion alters active Na^+^ transport across airway epithelial cells. Using radioactive tracers in combination with pharmacological inhibitors of ion transporters, we have also determined the mechanism of the change. Our results indicate that the ASLV expansion-induced changes in active Na^+^ transport reflect primarily an effect on the entry rather than the exit process.

## Materials and Methods

### Cell culture

Primary cultures of airway surface epithelium isolated from human or bovine trachea were grown as confluent monolayers on nucleopore cell culture inserts of 1 cm^2^ surface area, 0.45 *μ*m pore size, and 10 *μ*m thickness (Costar, Cambridge, MA) as described previously (Kondo et al. 1993; Yamaya et al. 1993; Yan et al. 2012). This cell culture insert system allows selective access to the basolateral and apical surfaces of the cells. Human tracheal epithelial cells (HTEC) and bovine tracheal epithelial cells (BTEC) were cultured under ALI with 1 mL of culture medium added only on the outside of the insert (basolateral side). Typically after 15–60 days of culture under basal ASLV conditions (ALI), cells become highly differentiated with a full active Na^+^ transport (Kondo et al. 1993; Yamaya et al. 1993; Yan et al. 2012). To study the effect of ASLV expansion on active Na^+^ transport mechanisms, 500 *μ*L of culture medium is added to the mucosal (Apical) surface of airway epithelial cells for the time period specified in each experiment.

### Measurement of monolayer bioelectric properties

To measure transepithelial electrical resistance (*R*_te_; Ω cm^2^) and the spontaneous transepithelial potential difference (p.d.; mV), we used a “chopstick” voltmeter (Millicell-ERS, Bedford, MA). To make the measurements on tissue grown under ALI, prewarmed and preoxygenated culture medium (500 *μ*L) was first added to the mucosal side of insert. Recordings were made within a minute of removing the cells from the CO_2_ incubator, and the added mucosal medium was removed immediately after the measurement. *R*_te_ estimates were corrected for the resistance of insert and medium alone (130 Ω cm^2^). The equivalent short-circuit current (*I*_eq_; *μ*A cm^−2^) was the ratio of p.d. and corrected *R*_te_ (Ohm's law). To monitor Na^+^ transport (*I*_sc_; *μ*A cm^−2^), cell sheets were mounted in conventional Ussing chambers and bathed in bicarbonate-buffered Krebs-Henseleit solution (pH 7.4) bubbled with 95% O_2_-5% CO_2_ at 37°C. Tissues were short-circuited with a voltage clamp (Model 762; Department of Bioengineering, University of Iowa City, IA) and transepithelial conductance (*G*_te_) was measured at 5-sec intervals from the deflections in current caused by constant voltage pulses (500 msec duration, 0.5–2 mV). After mounting, we waited few minutes to allow the baseline *I*_sc_ and *R*_te_ to stabilize. Drugs were then added as aliquots of 100- or 1000-fold concentrated stock solutions.

### Cellular ATP measurements

ATP concentration in HTEC or BTEC was quantified using an ATP bioluminescent assay kit as described elsewhere (Whitcutt et al. 1988). Briefly, cells were washed twice with cold PBS and incubated for 1 min at 4°C with 100 *μ*L of ice-cold perchloric acid (6%) added to the apical side. The cells were then homogenized for 30 sec in ice, and homogenates were centrifuged at 4°C at 581 *g* for 5 min. In an ice bath, supernatants were neutralized with NaOH and imidazole, centrifuged again at 4°C at 581 *g* for 1 min, then stored at −80°C until analysis (within 48 h). About 10 *μ*L of each sample was diluted in 0.55 mL of deionized water and 100 *μ*L was mixed with 100 *μ*L of luciferase reagent prewarmed at room temperature for 2.5 min. Luminescence was measured for a period of 10 sec. The protein content of each sample was determined using bicinchoninic acid (Smith et al. 1985). ATP levels were expressed as nmoles per mg protein.

### [^3^H]-ouabain-Na^+^/K^+^-ATPase pumps-binding assay

[^3^H]-ouabain binding was measured as described elsewhere (Widdicombe et al. 1979). Briefly, HTEC or BTEC was incubated for 2 h at 37°C in the CO_2_ incubator with 3 × 10^−8 ^mol L^−1^ of [^3^H]-ouabain (1 Ci per L, 43,000 Ci per mole, Amersham, UK) added in the basolateral compartment of cell culture inserts. Various amounts of nonradioactive ouabain (Sigma Chemicals, Saint Louis, MO) were added to the basolateral side to give a concentration range for ouabain from 3 × 10^−8 ^mol L^−1^ to 3 × 10^−4^ mol L^−1^. At the end of incubation, the culture inserts were washed for 15 sec in ice-cold physiologic saline (250 mL). Filters with their attached cells were cut from the insert and dissolved in 1 mL of 0.1 N NaOH (1 h, 60°C). Aliquots (50 *μ*L) of the NaOH solution were taken for protein determination using bicinchoninic acid (Smith et al. 1985). The remaining 950 *μ*L were treated with 50 *μ*L of glacial acetic acid (to minimize photo- and chemi-luminescence) and counted on a scintillation counter. Samples of incubation solution (50 *μ*L) were also counted after addition of NaOH and acetic acid as above. The plots of binding against concentration were fitted (Sigmaplot software, Systat Software Inc., San Jose, CA) according to *U* = *U*_max_(*X*/(*X* + *K*_d_)) + *aX*; Where *X* is the concentration of ouabain; *U*_max_ is the maximal specific-binding capacity of [^3^H]-ouabain; *K*_d_ is the dissociation constant of ouabain, and “*a*” describes nonspecific-binding depending linearly on ouabain concentration. Ouabain binding (*U*) was expressed as molecules per mg protein.

### Apical ^22^Na^+^ Uptake assay

^22^Na^+^ uptake was measured as described elsewhere (Frank et al. 2003). Ouabain (10^−5 ^mol L^−1^) and bumetanide (5 × 10^−5 ^mol L^−1^) were added to the basolateral culture medium of each culture insert for 5 min at 37°C in the cell incubator. Then, 0.2 *μ*Ci *μ*L^−1^ of ^22^Na^+^ (Amersham, UK) was added to the mucosal side in 0.2 mL of culture medium in the presence or absence of amiloride (10^−5^ mol L^−1^). After 5 min, the filters with attached cells were extensively rinsed in ice-cold physiologic saline, and lysed in 0.1 N NaOH. The protein and radioactivity counts were determined in the cell lysates, and ^22^Na^+^ uptake was reported as nEq per mg protein. All chemicals and reagents are from Sigma unless otherwise stated.

### Statistics

Results are presented as means ± SE. Tests of significant difference between means were performed by unpaired Student's *t*-test (*P* < 0.05 was considered significant).

## Results

**Figure 1:**
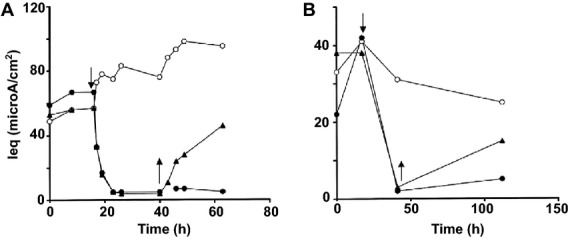
(A) Effect of ASLV expansion on equivalent short-circuit current (*I*_eq_) of bovine tracheal epithelial cells. Each time point represents the mean of *n* = 5–8 measurements from two cell cultures. SE of the mean values were below 4–12%. (B) Effect of ASLV expansion on equivalent short-circuit current (*I*_eq_) of human tracheal epithelial cells. Each time point represents the mean of *n* = 3 measurements from a single cell culture. Similar results were obtained in two other experiments. SE of the mean values were between 3 and 15%. For (A and B): *Open circles*-tissues maintained under ALI (basal ASLV: no medium added to apical side); *Closed circles*-tissues initially maintained under ALI, and then switched to expanded ASLV condition (500 *μ*L of medium added to apical side at the downward arrow); *Closed triangles*-tissues initially under ALI, switched to expanded ASLV condition at the downward arrow, and returned to ALI at the upward arrow.

**Figure 2:**
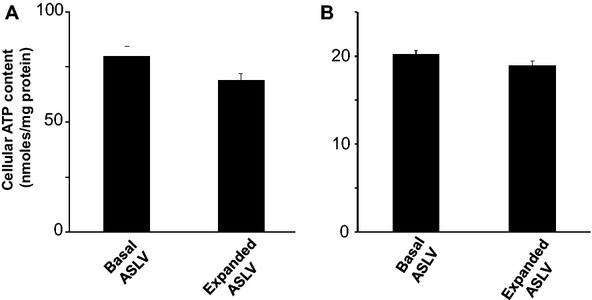
Effect of ASLV expansion on ATP content of bovine and human tracheal epithelial cells. (A) Bovine cells (ASLV expansion for 24 h); The tissues used had the following *I*_sc_ before ATP measurements (*μ*A cm^−2^; Mean ± SE; *n* = 4), basal ASLV: 84.8 ± 8.16, expanded ASLV: 9.97 ± 0.9. (B) Human cells (ASLV expansion for 48 h); The tissues used had the following *I*_sc_ before ATP measurements (*μ*A cm^−2^; Mean ± SE; *n* = 4), basal ASLV: 20.65 ± 0.25; expanded ASLV: 9.07 ± 0.69. Values are means ± SE of *n* = 4 measurements from a single cell culture. Similar results were obtained in two other experiments. The differences between ATP levels of basal ASLV and expanded ASLV in bovine or human cells are not statistically significant.

Airway surface liquid volume expansion reduced *I*_eq_ of bovine tracheal epithelium to 10% of baseline with a *t*_1/2_ of 1 h (Fig. 1A). If after 24 h of ASLV expansion, the mucosal medium was removed, then *I*_eq_ recovered with a *t*_1/2_ of 5–10 h. ASLV expansion had similar effects on *I*_eq_ of human tracheal cultures (Fig. 1B), though recovery of *I*_eq_ on return to basal ASLV (ALI) was slower than for bovine cells. In conventional Ussing chamber studies, as expected the addition of 10 *μ*mol L^−1^ amiloride inhibited baseline *I*_sc_ by 80%, consistent with sodium absorption that is mediated by ENaC channels. ASLV expansion for 24 h reduced baseline *I*_sc_ and *R*_te_ by about 90% and 50%, respectively (Table 1). Surprisingly, following ASLV expansion, amiloride had virtually no effect on *I*_sc_. This indicates that the decrease in *I*_sc_ caused by ASLV expansion reflects a decline in conductive ENaC-dependent sodium absorption. To further characterize the relationship between ASLV expansion and active sodium transport components, we reasoned that if ASLV expansion alters ATP level or sodium pumps function, then this will lead to reduction in apical sodium absorption (*I*_sc_). ASLV expansion did not significantly alter ATP levels in bovine (Fig. 2A) and human (Fig. 2B) tracheal cultures. Next, we examined binding of tritiated ouabain to bovine and human tracheal cultures under basal (ALI) and expanded ASLV conditions. Ouabain binds very specifically and irreversibly to the catalytic subunit of the Na^+^/K^+^-ATPase pumps in a 1:1 molar ratio (Ernst and Mills 1980). Moreover, ouabain binding to sodium pumps is strictly dependent on enzyme activity and presence of ATP which supports the phosphorylation of the enzyme and its turnover (Widdicombe et al. 1979; Ernst and Mills 1980).

**Table 1:** Effect of ASLV expansion on bioelectrical properties of bovine tracheal epithelial cells

ASLV	Basal		Expanded	
Am	−	+	−	+
*I*_sc_	72.0 ± 4.7	14.5 ± 0.4	7.3 ± 0.8	6.5 ± 0.7
*R*_te_	140.0 ± 16.5	167 ± 12.7	69.0 ± 13	72.0 ± 10.7

ASLV: airway surface liquid volume which represents the volume of culture medium added to apical side for 24 h (basal: no added medium, expanded: 500 *μ*L of added medium); Am: amiloride (10^−5^ mol L^−1^) added to mucosal side; *I*_sc_: short-circuit current (*μ*A cm^−2^); *R*_te_: transepithelial electrical resistance (Ω cm^2^); Means ± SE; *n* = 4 measurements.

**Figure 3:**
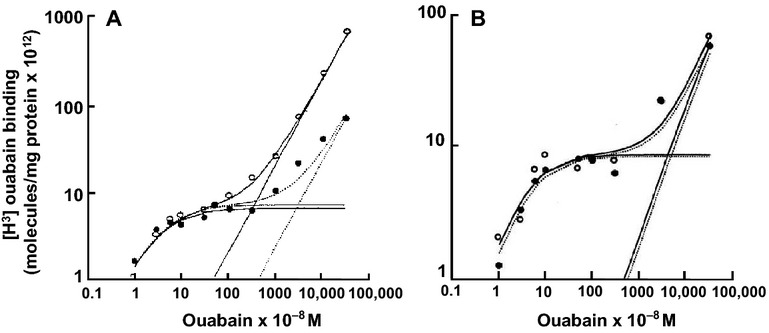
Effect of ASLV expansion on binding of [^3^H] ouabain to bovine and human tracheal epithelial cells. (A) Bovine cells (ASLV expansion for 24 h). (B) Human cells (ASLV expansion for 48 h). *Open circles & solid lines* – basal ASLV (ALI); *Closed circles & hatched lines* – expanded ASLV. The three curves for each set of points are saturable, nonsaturable and total binding. Single values from an experiment representative of three. Note that both ordinate and abscissa are logarithmic.

Binding of tritiated ouabain to bovine (Fig. 3A) and human (Fig. 3B) tracheal cultures exhibited a classic saturable component obeying Michaelis-Menten kinetics and a linear nonsaturable component when fitted to the equation described in materials and methods. The *K*_d_ for binding (40 nmol L^−1^), the maximal saturable binding (8 × 10^12^ molecules mg protein^−1^), and the nonspecific binding (20 × 10^16^ molecules mg protein^−1^) were not significantly different between basal (ALI) and expanded ASLV conditions (Fig. 3 and Table 2). Thus, the kinetics of tritiated ouabain binding to Na^+^/K^+^-ATPase pumps were not altered by ASLV expansion. Surprisingly, however, the nonspecific binding in bovine tracheal cultures was 10-fold higher under expanded than basal ASLV conditions (Fig. 3A and Table 2).

**Table 2:** Effect of ASLV expansion on kinetics of [^3^H]-ouabain binding to bovine and human tracheal epithelial cells

ASLV	Bovine		Human	
	Basal	Expanded	Basal	Expanded
*U*_max_	6.3 ± 0.5	6.9 ± 1.0	9.7 ± 1.6	9.5 ± 1.6
*K*_d_	3.6 ± 0.9	3.8 ± 0.5	3.8 ± 0.2	4.4 ± 0.6
*a*	22 ± 2.0	210 ± 22	24 ± 2.7	20 ± 2.6

ASLV: airway surface liquid volume which represents the volume of culture medium added to apical side for 24 h (bovine) or 48 h (human) (basal: no added medium, expanded: 500 *μ*L of added medium). *U*_max_: maximal specific-binding capacity of saturable [^3^H]-ouabain binding (×10^12^ molecules per mg protein); *K*_d_: dissociation constant (×10^−8 ^mol L^−1^) of [^3^H]-ouabain; a: nonspecific binding of [^3^H]-ouabain (×10^16^ molecules per mg protein). Values are means ± SE; *n* = 3 measurements.

**Figure 4:**
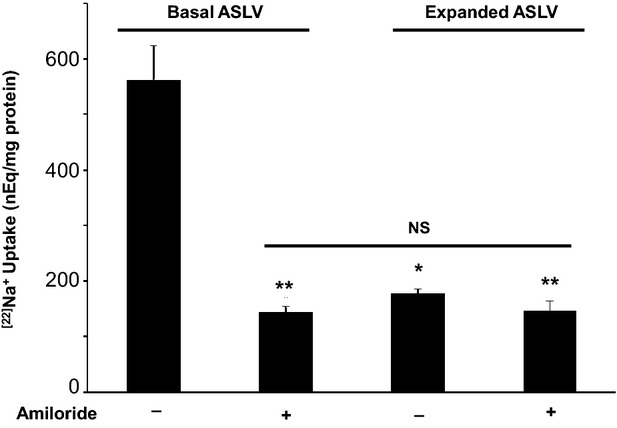
Effect of ASLV expansion on Uptake of ^22^Na^+^ across apical membrane of bovine tracheal epithelial cells. Ouabain (10^−5 ^mol L^−1^) and bumetanide (5 × 10^−5 ^mol L^−1^) were both added to the basolateral side for 5 min followed by apical ^22^Na^+^ Uptake for another 5 min. Amiloride: 10^−5 ^mol L^−1^ was added to mucosal medium. Values are means ± SE; *n* = 3 from one representative experiment. **Significantly different from control (*P* < 0.01). *Significant difference between basal and expanded ASLV (*P* < 0.05). NS: The differences between these conditions are not statistically significant. The tissues used had the following *I*_sc_ (*μ*A cm^−2^; Mean ± SE; *n* = 3) just before label uptake experiments, basal ASLV/amiloride^−^ (control): 83 ± 2; basal ASLV/amiloride^+^:76 ± 3; expanded ASLV/amiloride^−^: 7.7 ± 0.87; expanded ASLV/amiloride^+^: 11.7 ± 0.33.

To investigate the effect of ASLV expansion on apical sodium absorption, Uptake of ^22^Na^+^ was measured in bovine tracheal epithelium treated with ouabain. Under these conditions, the uptake of ^22^Na^+^ is representative of an increase in intracellular Na^+^ concentration (Taub and Saier 1979; Frank et al. 2003). Bumetanide was added to prevent net Na^+^ influx through the Na^+^/K^+^/2Cl^−^ cotransporter. As shown in Fig. 4, ASLV expansion reduced the amiloride-sensitive ^22^Na^+^ uptake by more than 70%, and caused a decrease of similar magnitude (90%) in *I*_sc_. However, ASLV expansion did not affect the level of residual amiloride-insensitive ^22^Na^+^ uptake.

## Discussion

It is well known that maintaining an adequate depth of ASL is important for airway epithelium ultrastructure and transepithelial ion transport (Van Scott et al. 1988; Kondo et al. 1991, 1993; Yamaya et al. 1992; Johnson et al. 1993). In this study, we have investigated how quickly the changes in airway epithelium bioelectrical properties occur in response to ASLV expansion. As in previous studies, bovine and human tracheal epithelial cells grown under ALI (basal ASLV) had high baseline *I*_eq_ of 30–100 *μ*A (Yamaya et al. 1992, 1993; Kondo et al. 1993). ASLV expansion inhibited the amiloride-sensitive *I*_sc_ by 90% with a *t*_1/2_ of 1 h indicating that ENaC-mediated sodium absorption was compromised (Fig. 1, Table 1). Of note, a similar slow decline in apical ENaC-mediated Na^+^ absorption reflected by a half-time of *I*_sc_ decay of 4 h was observed when channel biosynthesis is inhibited with cycloheximide in a kidney epithelial cell line (Butterworth et al. 2005). The decrease in airway epithelial cells resistance by 50% due to ASLV expansion can be attributed to alteration of epithelial tight junction structures which are essential for airway epithelium barrier function. In fact, airway epithelium integrity is necessary for proper ASL clearance, and when altered it leads to persistent airway edema (Matthay et al. 2002; Zemans and Matthay 2004; Mutlu and Sznajder 2005). Moreover, a positive correlation between the rate of ASL clearance and expression level of the tight junction protein claudin-4 was reported in human lungs (Wray et al. 2009; Rokkam et al. 2011). ASLV expansion up to 300 *μ*L (3 mm depth) for 24 h did not cause any significant effect on the bioelectrical properties of bovine cells (data not shown). Unfortunately, we did not test human cells but it is clear that they are more resistant to ASLV expansion than bovine cells even at a depth of 5 mm. As reported in Fig. 1, the time required for a maximum effect on bioelectrical properties of human cells is almost double the time needed for bovine cells in response to ASLV expansion by 500 *μ*L. Perhaps this initial resistance to ASLV expansion can be due to dilution of endogenous protease inhibitors as reported elsewhere (Myerburg et al. 2006; Tan et al. 2011). Especially, in the study of Tan and coworkers (Tan et al. 2011), ASLV expansion by 500 *μ*L increased *I*_eq_ by 50% within 1 h in human H441 airway epithelial cell line. Similarly we often observed a moderate increase of *I*_eq_ by about 20% in human tracheal epithelial cells after 1–7 h of ASLV expansion. However, this process was never been observed with bovine tracheal epithelial cells. To identify the pathways responsible for the decline in *I*_sc_ caused by ASLV expansion, we first measured cellular ATP concentration which is required for active sodium transport. In fact, reduced intracellular ATP (hypoxia) leads to reduced binding of ouabain to sodium pumps (Mills and Ernst 1975; Mills et al. 1977; Widdicombe et al. 1979). Moreover, the number and activity of sodium pumps (assessed by Michaelis–Menten kinetics binding of tritiated ouabain, Fig. 3) at the basolateral membrane of BTEC and HTEC were unchanged by ASLV expansion, and therefore do not account for the large decrease in short-circuit current. These results indicate that ASLV expansion for 24 h (bovine cells) or 48 h (human cells) did not affect significantly cell oxidative metabolism but we cannot exclude the possibility that cells may become hypoxic at a later time. The [^3^H]-ouabain-binding assay used in this study provides a reliable determination of the number of functional sodium pumps (Widdicombe et al. 1979). Indeed, a strong correlation between ouabain-sensitive Rb^+^ uptake and tritiated ouabain-binding was reported in Xenopus laevis oocytes (Schmalzing et al. 1991). However, ouabain uptake does occur via internalization of functional Na^+^ pumps (Schmalzing et al. 1989; Yan et al. 2012; Cherniavsky-Lev et al. 2014). Therefore, the [^3^H]-ouabain-binding assay may disclose a fraction of intracellular functional sodium pumps which leads to an overestimation of their surface density. A surprising finding in our ouabain-binding studies on bovine cultures was the 10-fold increase in the nonspecific uptake of label induced by ASLV expansion. The mechanism is unknown, but given the short exposure time, it is unlikely to represent major tissue remodeling. One possibility is that ASLV expansion stimulates pinocytosis. The same effect was not seen in human cell cultures, but these have a very different structure from bovine. Human cells consist of two or three layers of cells of 15 *μ*m total thickness (Yamaya et al. 1993); Bovine cultures have multiple cell layers up to 100 *μ*m or more in total depth (Kondo et al. 1993).

In apparent contrast to our present study, Azzam and coworkers have reported that SHP elevation to 15 cm H_2_O for 60 min was associated with reduction of Na^+^ pumps activity and protein level (Azzam et al. 2002). However, it is unclear why they reported a similar inhibitory effect of ouabain on ASL clearance at elevated SHP compared to control. Also, a potential weakness of their results is that they carried out in vitro measurements of Na^+^ pumps in basolateral membrane samples isolated from peripheral lung tissue, which include membranes from alveolar epithelial and endothelial cells. This heterogeneity in cells may well underestimate the number and activity of sodium pumps in airway epithelial cells according to the authors (Azzam et al. 2002). Therefore, this situation is markedly different from our present study, where we used intact primary cultures of airway epithelial cells that contain one type of cells, and the effect of ASLV expansion rather than SHP was evaluated.

Airway surface liquid volume expansion dramatically reduced the amiloride-sensitive component of ^22^Na^+^ uptake by more than 70%, which correlated with a strong decrease in amiloride-sensitive short-circuit current (85%). Conversely, levels of residual amiloride-insensitive ^22^Na^+^ uptake under basal and expanded ASLV were very similar and may well correspond to sodium absorption through the nonselective cyclic nucleotide-gated (CNG) cation channels (Zemans and Matthay 2004; O'Brodovich et al. 2008; Wilkinson et al. 2011). Indeed, a residual amiloride-insensitive ^22^Na^+^ uptake component was previously measured in bovine tracheal epithelium treated with amiloride (Langridge-Smith 1986). Interestingly Sugita et al. (2003) found that lung perfusion for 4 or 8 h after transplantation reduced levels of ENaC *α*- and *β*- subunits mRNA by 75% and 50% respectively, and the protein level of ENaC *α*-subunit was also decreased by 75%. In contrast, mRNA levels of Na^+^ pumps *α*_1_/*β*_1_- subunits were unchanged though they did not measure their protein level. Another animal study that also corroborates our findings was conducted on ex vivo rat lungs submitted to a left atrial pressure (LAP) of 15 cm H_2_O (Kaestle et al. 2007). Kaestle and co-workers found that impairment of fluid reabsorption by high SHP is due to alteration of apical Na^+^ channels but not Na^+^ pumps functions.

Our study did not address the mechanism by which amiloride-sensitive apical Na^+^ uptake was altered, but given the magnitude of the change, it seems probable that ASLV expansion reduced numbers of functional Na^+^ channels in the apical membrane. Whether this is due to a change in the probability of opening (*P*O) of individual channels or a reduction in the numbers of channels per unit area of membrane (*N*) is uncertain. Indeed, regulation of channel activity occurs mostly through alterations in either *N* or *P*O (Butterworth et al. 2009). Especially, apical membrane channel number is mostly controlled by exocytosis/endocytosis cycles of vesicles from subapical locations (Loo et al. 1983; Butterworth et al. 2009; Edinger et al. 2012) but it can also be regulated by increase in synthesis and delivery of channel subunits (Butterworth et al. 2009). The recovery of *I*_eq_ on return of cells to ALI (basal ASLV) had a *t*_1/2_ of 5–10 h. This is slow enough to represent the de novo synthesis and insertion of apical membrane Na^+^ channels.

In our previous study (Azizi et al. 1997), we did not observe any notorious direct effect of elevated SHP, up to 20 cm H_2_O, on *I*_sc_ (active ion transport), but the present experiments revealed a surprising and striking effect of ASLV expansion on ENaC-mediated sodium absorption regardless of the cause or origin of ASLV expansion. However, other factors (e.g., inflammatory mediators) that are released in vivo during airway inflammation may also contribute to alteration of ENaC-mediated sodium absorption. For example, it was shown that TGF-*β*1 decreased apical amiloride-sensitive ^22^Na^+^ uptake in both rat and human alveolar type (ATII) cells due to decreased expression of *α*-ENaC subunit mRNA and protein levels (Frank et al. 2003). Recently, another study showed that TGF-*β* treatment reduced alveolar liquid clearance by 50% and ^22^Na^+^ uptake by 80% in rabbit lungs (Peters et al. 2014). Interestingly, TGF-*β* was found to target the amiloride-sensitive Na^+^ channels, but Not the Na^+^ pumps in lung epithelial cells (A549) and primary mouse ATII cells. Moreover, TGF-*β* did not alter steady-state mRNA levels of ENaC subunits but drove a rapid internalization and reduction of cell-surface ENaC channels (Peters et al. 2014). TGF-*β*, like ASLV expansion, affected amiloride-sensitive Na^+^ absorption within hours and with similar magnitude, and therefore it is tempting to speculate that inhibition of apical Na^+^ uptake by ASLV expansion might be due in part to internalization of apical ENaC channels and subsequent decrease of their cell-surface numbers.

In summary, active amiloride-sensitive Na^+^ transport across airway epithelium is known to underlie the absorption of salt and water (Smith and Welsh 1992; Jiang et al. 1993; Zabner et al. 1998) and thereby regulates the depth (Wu et al. 1998) and possibly the ion content (Widdicombe 1999) of the airway surface liquid. Here, we show that when the liquid on the mucosal surface of airway epithelium is increased beyond the normal “physiological” level, then active absorption of Na^+^ is inhibited and airway epithelium integrity is altered. Clinically, this will retard or impair the clearance of liquid from flooded alveoli and small airways, and therefore contribute to the pathophysiology of hydrostatic pulmonary edema. The findings of this study, if confirmed in vivo, would constitute a shift in the current paradigm of pulmonary edema development and resolution mechanisms, with profound implications for the way therapeutic interventions are undertaken to treat pulmonary edema. Independent of its cause or origin, ASLV expansion is an important factor that has to be carefully taken into consideration during any pharmacological approach that targets airway epithelium amiloride-sensitive Na^+^ channels for the treatment of pulmonary edema. A good example is the *β*2-adrenergic receptor agonist (e.g., Salbutamol or Albuterol) that stimulates recruitment or de novo synthesis of amiloride-sensitive Na^+^ channels (Fronius 2013). It was ineffective as a treatment of pulmonary edema in two relatively recent multicenter clinical trials in critically ill patients either with acute lung injury (Matthay et al. 2011) or ARDS (Gao Smith et al. 2012) despite evidence of beneficial effect observed in cell culture as well as animal studies (Fronius 2013). It might be considered that accumulation of liquid in the airways (e.g., alveolar airspace) would take part in preventing the therapeutic effect of *β*2-adrenergic receptor agonists in patients with severe acute lung injury or ARDS. Therefore, it makes sense to consider strategies that are complementary and follow each other in time in order to reduce lung water content and make ENaC-targeted drugs more effective in restoring airway epithelium integrity and active ion transport.
